# Robust Expression and Secretion of Xylanase1 in *Chlamydomonas reinhardtii* by Fusion to a Selection Gene and Processing with the FMDV 2A Peptide

**DOI:** 10.1371/journal.pone.0043349

**Published:** 2012-08-24

**Authors:** Beth A. Rasala, Philip A. Lee, Zhouxin Shen, Steven P. Briggs, Michael Mendez, Stephen P. Mayfield

**Affiliations:** 1 The San Diego Center for Algae Biotechnology and Division of Molecular Biology, University of California San Diego, San Diego, California, United States of America; 2 Sapphire Energy, San Diego, California, United States of America; East Carolina University, United States of America

## Abstract

Microalgae have recently received attention as a potential low-cost host for the production of recombinant proteins and novel metabolites. However, a major obstacle to the development of algae as an industrial platform has been the poor expression of heterologous genes from the nuclear genome. Here we describe a nuclear expression strategy using the foot-and-mouth-disease-virus 2A self-cleavage peptide to transcriptionally fuse heterologous gene expression to antibiotic resistance in *Chlamydomonas reinhardtii*. We demonstrate that strains transformed with *ble-2A-GFP* are zeocin-resistant and accumulate high levels of GFP that is properly ‘cleaved’ at the FMDV 2A peptide resulting in monomeric, cytosolic GFP that is easily detectable by in-gel fluorescence analysis or fluorescent microscopy. Furthermore, we used our ble2A nuclear expression vector to engineer the heterologous expression of the industrial enzyme, xylanase. We demonstrate that linking *xyn1* expression to *ble2A* expression on the same open reading frame led to a dramatic (∼100-fold) increase in xylanase activity in cells lysates compared to the unlinked construct. Finally, by inserting an endogenous secretion signal between the *ble2A* and *xyn1* coding regions, we were able to target monomeric xylanase for secretion. The novel microalgae nuclear expression strategy described here enables the selection of transgenic lines that are efficiently expressing the heterologous gene-of-interest and should prove valuable for basic research as well as algal biotechnology.

## Introduction

Microalgae are a diverse group of photosynthetic microorganisms with considerable biotechnological potential. Algal products are currently used in the animal and fish feed industries, and for cosmetics, pigments, and nutraceuticals [1]–[3]. Furthermore, microalgae have the potential to be a valuable source of bioenergy [4]–[6]. Genome engineering in algae offers the potential for improved product yields and crop protection, as well as the potential to modify metabolic pathways to produce unique products [7]–[10]. Importantly, transgenic microalgae also have the potential to be low-cost bioreactors for commercially valuable recombinant proteins such as therapeutic proteins and industrial enzymes [11]–[14]. However, genetic engineering of microalgae is still far behind other microorganisms. A major obstacle remains low transgene expression levels from the nuclear genome of many microalgae. Here, we report the robust expression and secretion of a commercially valuable industrial enzyme, xylanase, from the nuclear genome of the microalga *Chlamydomonas reinhardtii* by linking the xylanase gene directly to an antibiotic resistance gene via the foot and mouth disease virus (FMDV) self cleaving 2A sequence.

*C. reinhardtii* is a freshwater, green microalga that has been a popular model organism for physiological, molecular, biochemical and genetic studies. As such, it has a well-developed molecular genetic toolkit [15]. *C. reinhardtii* has also gained attention as a platform for the production of therapeutic proteins and vaccines [12], [16], [17]. While genetic transformation techniques are well established for both the nuclear and chloroplast genomes in *C. reinhardtii*, only transgene expression in the chloroplast has led to protein accumulation to economically viable levels [16], [18]–[27]. However, transgene expression from the nuclear genome offers several advantages over chloroplast expression, such as glycosylation and other post-translational modifications and heterologous protein-targeting to sub-cellular locations or for secretion [28]. While the molecular mechanism(s) for poor transgene expression from the nuclear genome are not completely understood, possible reasons include poor promoters, genome integration position effects, and transgene silencing. Nuclear transformation occurs primarily by random insertion through non-homologous end joining [29]. This often leads to ‘position effect’, in which the level of transgene expression is influenced by the surrounding genomic regions [30]. A more significant roadblock to nuclear engineering in *C. reinhardtii* is transgene silencing. Reports have demonstrated transgene silencing at both the transcriptional and post-transcriptional levels [31]–[34]. Thus, it is necessary to screen large numbers of transformants to identify individual clones that demonstrate the desirable, or sometimes even detectable, level of protein expression.

Several advancements have been developed for improved nuclear transgene expression. Codon optimization of GFP was required for its expression in *C. reinhardtii* [35]. The insertion of endogenous intron(s) into transgenes and/or the surrounding 5′ and 3′ untranslated regions leads to improved expression [36], [37]. Choice of promoter has also been shown to have an effect. Expression from the endogenous rbcs2 and β-tubulin promoters are improved by fusion with the HSP70A promoter, which seems to act as an activator [38]–[41]. Fusion of GFP to the bleomycin/zeocin-resistance gene *sh-ble* was required to obtain detectable levels of GFP [35], and a ble-luciferase fusion was shown to support higher levels of luciferase expression compared to unfused luciferase [42]. The *sh-ble* gene product confers resistance to the DNA double strand break-inducing bleomycin family of antibiotics through binding and sequestration, thus antibiotic resistance is proportional to *sh-ble* expression levels [43]. Finally, UV mutagenesis of transgenic *C. reinhardtii* has been used to select for algal strains with improved transgene expression [44]. However, despite these notable advancements to nuclear transgene expression, recombinant protein accumulation still remains quite low, with the highest reported recombinant protein accumulation at 0.2% of total soluble protein [44]. This was achieved in a UV-mutagenized strain with unknown and unmapped mutation(s). Thus, the development of novel strategies for higher and more consistent transgene expression is needed, and should advance many areas of algal research and biotechnology.

Here, we explore the potential application of using the foot-and-mouth-disease-virus (FMDV) 2A peptide to link transgene expression to that of a selection marker in *C. reinhardtii*. The FMDV 2A peptide encodes a short ∼20 amino acid sequence that mediates a self-cleavage reaction [45]. It is believed that during translation elongation of the 2A sequence, a peptide bond fails to form between the last two amino acids of the 2A sequence [46]. Thus, when 2A is fused between two genes in a single open reading frame (ORF), the resulting protein is processed to yield two discrete proteins, with the short 2A sequence fused to the C-terminus of the first protein product. FMDV 2A and 2A-like sequences have been used for heterologous gene expression and biomedical applications in many eukaryotic systems including mammalian cell culture, retroviral gene therapy, and transgenic plants [47]–[49]. We constructed a *C. reinhardtii* nuclear expression vector in which *GFP* or *xylanase 1* (*xyn1*) from *Trichoderma reesei* was fused to the bleomycin antibiotic resistance gene *sh-ble* via the 2A sequence. We demonstrate the 2A peptide is correctly ‘processed’ in the algal cytoplasm, resulting in ‘cleavage’ of GFP or Xyn1 from Ble-2A. GFP and Xyn1 are stably expressed and functional. By linking the *xyn1* to *ble-2A* we could improve xylanase activity by approximately 100-fold compared to unlinked *xyn1* expression. Finally, by fusing an endogenous secretion signal between *ble-2A* and *xyn1*, we were able to target Xyn1 for secretion, resulting in significant accumulation of highly active xylanase enzyme in the culture media.

## Results

### Functional analysis of the 2A peptide in *C. reinhardtii*

**Figure 1:**
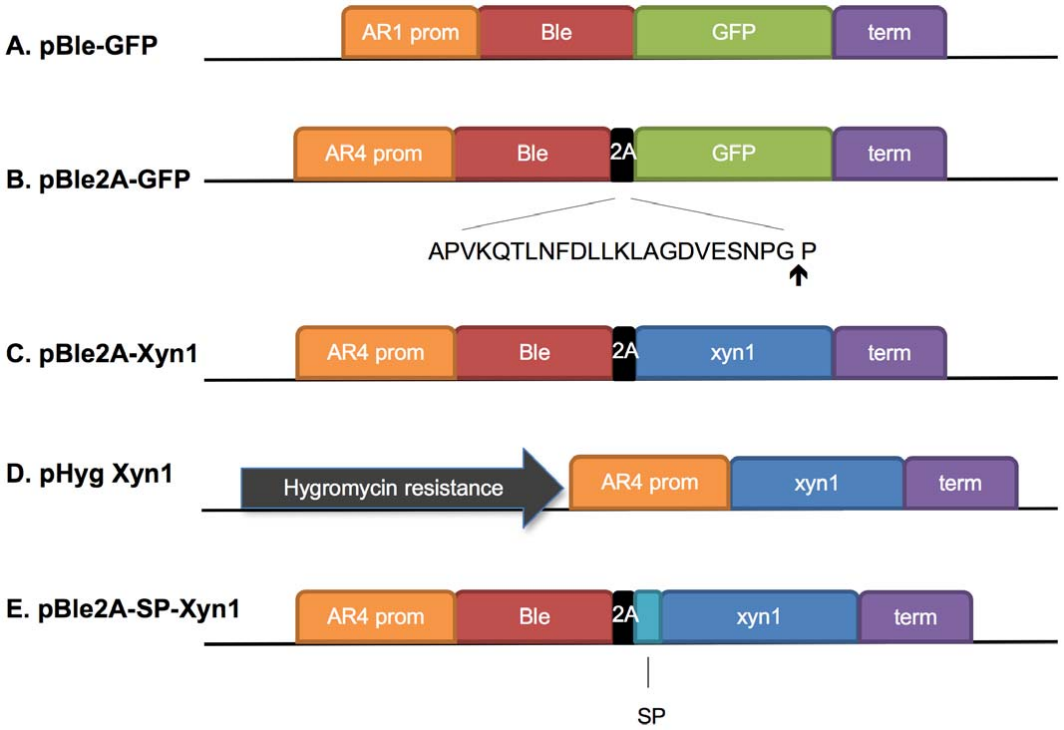
Constructs used for heterologous gene expression from the nuclear genome of *C. reinhardtii*. Vector maps represent the constructs used in the study including (A) P_AR1_::Ble-GFP, (B) P_AR4_::Ble-2A-GFP, (C) P_AR4_::Ble-2A-xyn1, (D) pHyg P_AR4_::Xyn1, and (E) P_AR4_::Ble-2A-SP-Xyn1. 2A, FMDV 2A peptide, ↑ designates the site of cleavage. Term, rbcs2 3′ terminator. Signal peptide (SP), *C.r. ars1* secretion signal sequence.

To determine whether the FMDV 2A ‘self-cleaving’ peptide could be expressed and is functional in *C. reinhardtii*, we constructed a nuclear transformation vector in which 2A was placed between the *sh-ble* bleomycin/zeocin-resistance gene [50] and nuclear codon-optimized *GFP* [35] (Figure 1B). The ble protein product (referred to as Ble in this study) binds zeocin in a 1∶1 ratio to prevent the antibiotic from inducing DNA double strand breaks [43]. Thus, unlike enzymatic antibiotic resistance genes, only transformants expressing *ble* to relatively high levels survive zeocin selection. Indeed direct fusions between Ble and GFP or Renilla luciferase led to improved expression of the reporter proteins compared to unfused constructs [35], [42]. Therefore we purposely chose the *ble* selection marker in this study in the hopes of only selecting for transgenic lines that express high levels of the linked gene of interest. The *ble-2A-gfp* ORF was placed under the control of the hsp70/rbcs2 promoter and 5′ UTR [39] which was modified to contain four copies of the first intron of rbcs2 between the hsp70A and rbcs2 promoters (P_AR4_) (Figure 1B). The *ble* gene was also interrupted with a copy of the first rbcs2 intron. The 2A vector was compared to the pBle-GFP direct fusion (Figure 1A), which leads to accumulation of functional Ble-GFP fusion protein in the nucleus [35]. The constructs were transformed by electroporation into the cell-wall deficient *C. reinhardtii* strain cc3395 [51]. Both constructs yielded transformation efficiencies of approximately 200 colonies per µg of DNA when selected on TAP/agar plates containing 15 µg/ml zeocin (data not shown). This suggests that the *ble2A-GFP* ORF leads to functional Ble protein product able to confer resistance to zeocin.

**Figure 2:**
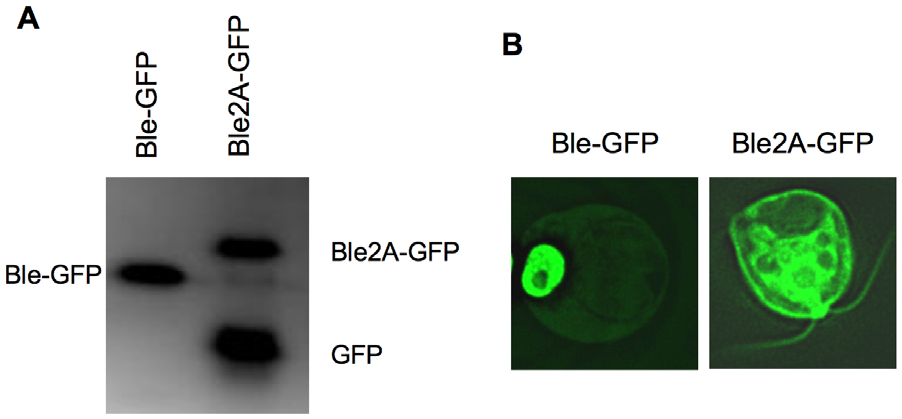
FMDV 2A peptide ‘cleavage’ in *C. reinhardtii*. A. SDS-PAGE in-gel fluorescence analysis of total protein isolated from transgenic lines expressing *ble-GFP* or *ble-2A-GFP*. Labeled bands represent in-gel fluorescence signals of the respective heterologous proteins. B. Microscopy images of GFP signals from transgenic cells expressing *ble-GFP* or *ble-2A-GFP*.

To determine whether the ble2A-GFP polyprotein was ‘cleaved’ in *C. reinhardtii*, in-gel fluorescence analysis was performed on lysates obtained from a representative Ble-GFP and Ble2A-GFP transgenic line (Figure 2A). As expected, a single fluorescent band was detected in Ble-GFP lysates, corresponding to the Ble-GFP fusion protein. However, the Ble2A-GFP lysates contained two fluorescent bands. Immunoblot analysis using an anti-GFP antibody indicated that the major band migrated at the expected size for GFP, while the minor band migrated at the expected size for unprocessed Ble2A-GFP (data not shown). Fluorescent microscopy was used to compare GFP localizations in these two lines. As previously demonstrated by Fuhrmann et al, 1999, the Ble-GFP fusion protein localizes to the nucleus (Figure 2B, left panel). However, in the Ble2A-GFP line, the GFP signal is found diffused through the cytoplasm (Figure 2B, right panel).

Together, these data demonstrate that the ble2A-GFP polyprotein is well expressed and the majority of the 2A sequence is properly ‘processed’ in *C. reinhardtii*, leading to two discrete and functional translation products, Ble2A and GFP.

### Expression of the industrial enzyme, *T. reesei xyn1*, in *C. reinhardtii* using FMDV 2A

To determine whether the Ble2A expression system could be used to express a commercially relevant industrial enzyme, we attempted to heterologously express xylanase 1 (Xyn1) from *Trichoderma reesei* [52]. *T. r.* Xyn1 is an endo-β-1,4-xylanase (EC 3.2.1.8) that functions in hemicellulose breakdown by hydrolyzing xylan and xylo-oligosaccharides [53]. Endoxylanases are important industrial enzymes used in a wide range of industries including baking, textiles, pulp and paper manufacturing, animal feed and could potentially be used in the future at large scale for cellulosic biofuel production [53], [54].

**Figure 3:**
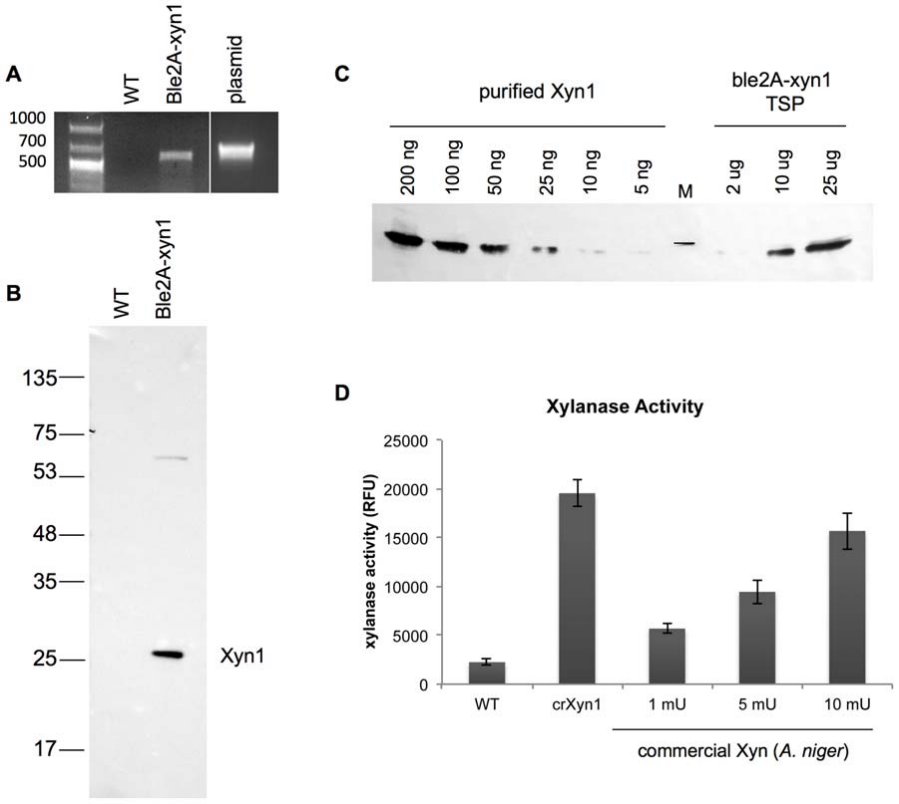
Expression and activity of *T. reesei* Xyn1 from the ble2A vector in *C. reinhardtii*. A. PCR analysis of whole cell lysates from the parental cc1690 (WT) and *ble2A-xyn1* strains with primers specific to *xyn1* reveals that the transformant is stably transformed. A plasmid containing *ble2A-xyn1* is included as a positive control. The expected size of the PCR product is 579 bps. B. Immunoblot analysis of 25 µg of total soluble protein isolated from cc1690 (WT), and a transgenic line transformed with *ble2A-xyn1*. C. Quantitative immunoblot of monomeric Xyn1. The indicated amount of total soluble protein from the ble2A-xyn strain (right) was compared against a dilution series of purified Xyn1 (left). Densitometric analysis of immunoblot signals using the ImageJ software indicates that Xyn1 accumulates to approximately 0.25% of total soluble protein. The marker (M) shown represents the 26 kDa marker protein. D. Quantitation of xylanase activity in the lysates of *C. reinhardtii*. Xylanase activity, as measured by the accumulation of the fluorogenic xylanase product DiFMU and represented in relative fluorescence units (RFU), in 50 µg of total soluble protein from the ble2A-xyn1 transgenic line (CrXyn1) is compared to 1 mU, 5 mU, and 10 mU of commercially available Xylanase (*A. niger*, Megazyme). WT (cc1690) lysate is included as a negative control. Hydrolysis reactions were allowed to proceed for 5 minutes and fluorescence was measured in a Tecan microplate reader (ex360 nm/em460 nm). Data points represent averages of triplicate measurements with error bars representing ± one standard deviation (SD).

To enable heterologous expression of *T. reesei xyn1* in *C. reinhardtii*, we first synthesized the gene according to the microalga's nuclear codon bias. A metal affinity tag (MAT) and 1× FLAG epitope tag was added to the C-terminus for ease in identification and purification. Codon-optimized Xyn1 was cloned into the Ble2A expression vector (Figure 1C) and transformed into wild type cc1690 by electroporation. Transformants were selected on TAP/agar plates supplemented with 10–15 µg/ml zeocin. Zeocin resistant clones were screened for Xyn1 expression using dot blots, and a representative clone was chosen for further analysis. To confirm that the Ble2A-Xyn1 clone was indeed stably transformed with *xyn1*, the clone was analyzed by PCR using oligonucleotides specific to *xyn1*. A band corresponding to the predicted size of 579 bps was seen in PCR reactions on the plasmid pBle2A-xyn1 and on Ble2A-Xyn1 cell lysate, but not on wild type cc1690 lysate (Figure 3A). Immunoblot analysis of 25 µg of total soluble protein from the Ble2A-Xyn1 transgenic line reveals that Xyn1 is well-expressed (Figure 3B). By far the major band identified runs at the predicted molecular weight of Xyn1, 26 kDa, indicating that a majority of the Ble2A-Xyn1 polyprotein is ‘processed’ to yield monomeric Xyn1 (Figure 3B). The unprocessed Ble2A-xyn1 protein product, which has a predicted molecular weight of 42.7 kDa, was not detected. However, we did observe a band at approximately 60 kDa, which may be aggregated xylanase. Quantification of monomeric Xyn1 accumulation against a dilution series of purified Xyn1 revealed that the enzyme accumulated to approximately 0.25% of total soluble protein (Figure 3C).

The activity of algal-expressed Xyn1 was compared to commercial *Aspergillus niger* Xylanase (Megazyme) using the fluorescence-based EnzChek® Ultra Xylanase Assay Kit (Invitrogen, Carlsbad, CA). Hydrolysis of the substrate, 6,8-difluoro-4-methylumbelliferyl β-d-xylobioside (DiFMUX_2_), to fluorescent DiFMU corresponds to xylanase activity and can be monitored at excitation 385 nm/emission 455 nm [55]. 50 µg of total soluble protein from wild type cc1690 and the ble2A-xyn1 line were incubated in triplicate with the xylanase substrate and fluorescence readings were taken every 5 minutes for 20 minutes using a Tecan plate reader. 1 mU, 5 mU and 10 mU of *A. niger* Xylanase was also included in the assay in triplicate. 50 µg of total soluble protein from the ble2A-xyn1 line harbored more xylanase activity than 10 mU of commercial *A. niger* Xylanase (Figure 3D).

Thus, expression of Xyn1 from the Ble2A expression vector leads to the accumulation of monomeric and active enzyme in *C. reinhardtii*.

### Linking the *xyn1* gene to the *ble* selection marker through the 2A peptide significantly improves xylanase expression

**Figure 4:**
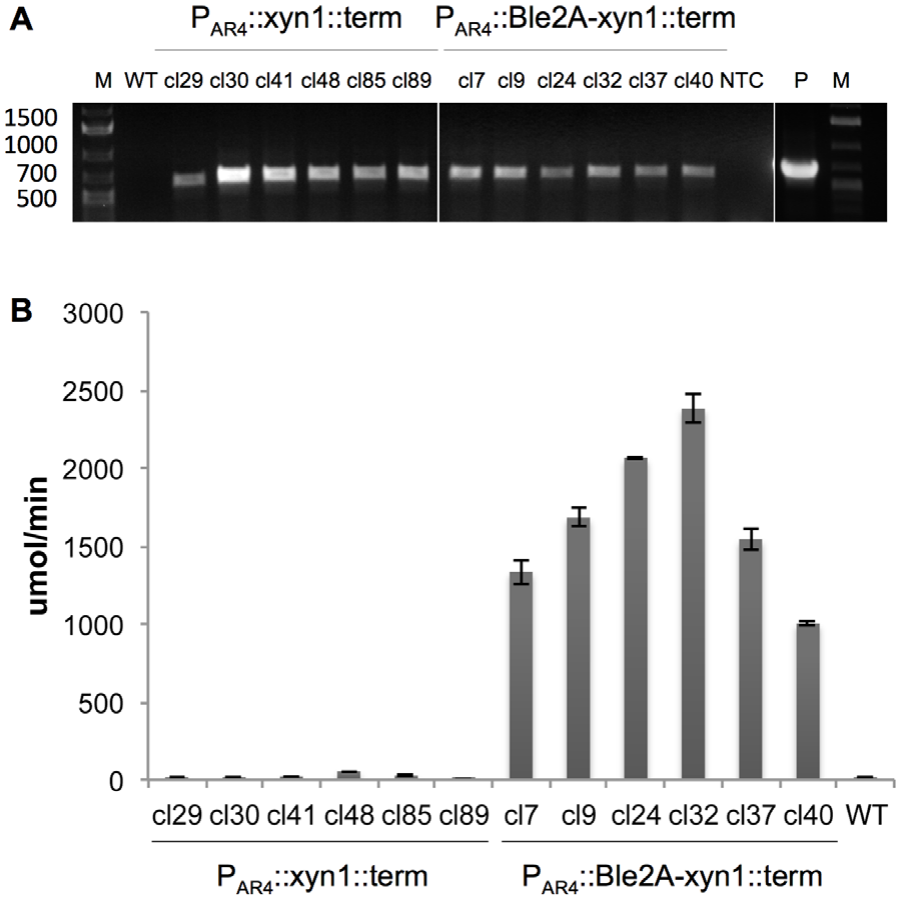
Co-expression of Xyn1 with Ble-2A leads to the selection of transformants with higher xylanase activity. A. PCR analysis for the presence of *xyn1*, in 6 independent clones (cl) transformed with *P_AR4_::xyn1* or *P_AR4_::ble2A-xyn1*. The expected size of the PCR product is 579 bps. WT, parental strain cc1690; NTC, no template control; P, plasmid positive control; M, 1 kb+ marker. B. Comparison of xylanase activities in 6 independent clones (cl) transformed with *xyn1* (left) or *ble-2A-xyn1* (right). Xylanase activity is represented as rate of accumulation of the fluorogenic xylanase product DiFMU in µmol/min. Xylanase reactions contain 50 µg of total soluble protein from each clone. Data points represent averages of triplicate measurements with error bars representing ± one SD. Wild type (WT, cc1690) is also included for comparison.

The nuclear genome of *C. reinhardtii* was first transformed with a heterologous gene in 1990 [56]. Since then, many other heterologous transgenes have been expressed, including antibiotic resistance markers and reporter genes. Generally, heterologous genes are placed under the control of endogenous regulatory elements, including a promoter and 5′ untranslated region (UTR), and 3′ terminator. The expression cassette is then co-transformed with a selection marker, either on the same plasmid or on separate plasmids. However, this strategy is plagued by position effect, low levels of expression, and transgene silencing. We wanted to compare the xylanase expression levels from our Ble2A expression vector to that of the traditional expression strategy, to determine the effect, if any, of linking *xyn1* expression to *ble* expression. Thus we cloned the codon-optimized *xyn1* into the pHyg vector (Figure 1D). This vector contains the hygromycin B resistance gene *aph7″* from *Streptomyces hygroscopicus* under the control of the endogenous β-tubulin promoter and 5′ UTR [57]. *Xyn1* was cloned behind the AR4 promoter (P_AR4_), which is identical to the promoter driving Ble2A-xyn1 (Figure 1C). Therefore, the pHygB xyn1 transformation vector represents the traditional method for heterologous gene expression from the nuclear genome of *C. reinhardtii*. Vectors containing P_AR4_::Ble2A-xyn1 and P_AR4_::xyn1 were linearized and electroporated into cc1690. P_AR4_::Ble2A-xyn1 transformants were selected on TAP/agar plates supplemented with 15 µg/ml zeocin, while the P_AR4_::xyn1 transformants were selected on TAP/agar plates supplemented with 15 µg/ml hygromycin B. 94 individual P_AR4_::xyn1 transformants were PCR-screened for the presence of the xyn1 gene: 55 clones (58%) were gene-positive. Likewise, 42 independent P_AR4_::Ble2A-xyn1 lines were PCR-screened for the presence of xyn1, and 30 (70%) were identified as being gene positive (data not shown). 26 and 24 gene positive P_AR4_::Ble2A-xyn1 and P_AR4_::xyn1 clones, respectively, were picked at random and screened for xylanase activity in a 96-well plate assay (data not shown). The best 6 xylanase-expressing clones for each construct were chosen for further analysis. Stable integration of *xyn1* was confirmed by PCR analysis on cell lysates (Figure 4A). The clones were inoculated into liquid TAP media and allowed to grow until mid-log phase. Cells were isolated, lysed by sonication, and the total soluble protein fraction was retained. Following protein quantitation by Bradford analysis, lysates were diluted to 1 µg/µl in xylanase assay buffer and 50 µg of each lysate was assayed in duplicate for xylanase activity. Wild type cc1690 lysate was included as a negative control. The results are summarized in Figure 4B. Five out of the six P_AR4_::xyn1 clones displayed very low, but detectable, levels of xylanase activity. However, the 6 P_AR4_::Ble2A-xyn1 lines contained comparably robust xylanase activity as measured by the rate of accumulation of the fluorescent product (µmol/minute) (Figure 4B). Indeed, we saw a ∼100 - fold increase in xylanase activity when *xyn1* was expressed from the P_AR4_::Ble2A-xyn1 construct compared to P_AR4_::xyn1. Thus, the data demonstrates that directly linking *xyn1* expression to the *ble* selection marker through the 2A peptide leads to significantly higher levels of expression compared to the unlinked construct.

### Ble2A expression system can be used to secrete enzymatically active Xyn1

One of the advantages of nuclear transgene expression is the ability to target heterologous protein to sub-cellular locations or for secretion. Targeting proteins to the secretory pathway allows for correct post-translational processing. Futhermore, secretion of recombinant proteins to the culture medium simplifies purification and downstream processing. To determine whether the Ble2A expression system can be used to target heterologous proteins for secretion in *C. reinhardtii*, we inserted the endogenous *ars1* secretion signal sequence (SP) between the *ble2A* and *xyn1* genes (Figure 1E). pBle2A-Xyn1 and pBle2A-SP-Xyn1 constructs were electroporated into the cell wall deficient strain cc3395. Transformants were selected on TAP/zeocin plates supplemented with 100 µg/ml arginine. Transformants were screened for the presence of *xyn1* by PCR and for xylanase activity using the EnzChek® Ultra Xylanase Activity Kit (data not shown). A representative Ble2A-Xyn1 and Ble2A-SP-Xyn1 line were chosen for further analysis.

**Figure 5:**
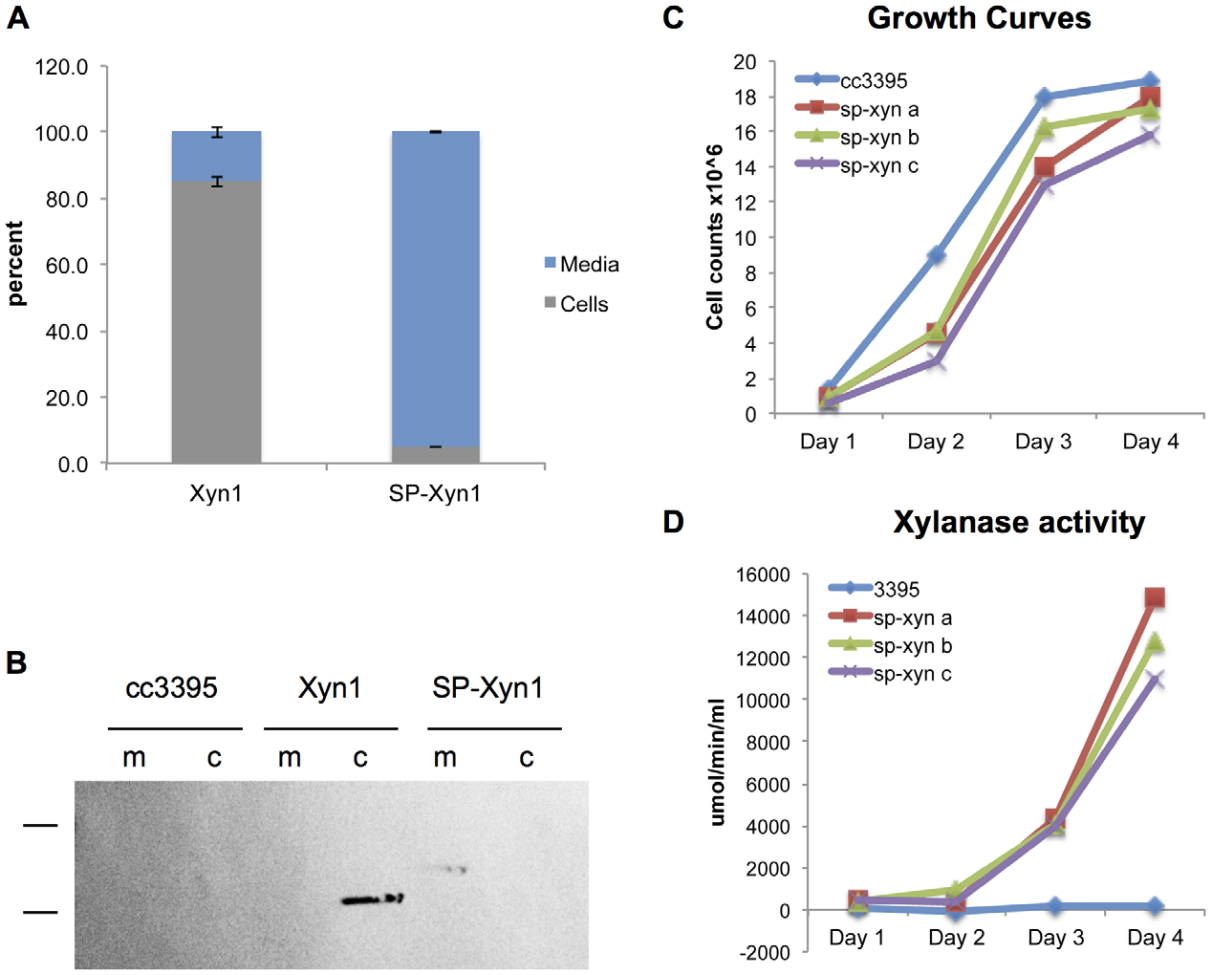
Insertion of a secretion signal peptide between FMDV 2A and Xyn1 leads to robust accumulation of functional Xyn1 in the culture media. A. Comparison of intracellular and extracellular xylanase activity for the *ble2A-xyn1* (Xyn1) and *ble2A-SP-xyn1* (SP-Xyn1) transgenic lines. Intracellular (cells, grey) and extracellular (media, blue) xylanase activities are represented as a percent of total activity for each strain. Both lines were grown in triplicate cultures and the data represents the average percentage of the triplicate cultures. Xylanase reactions were allowed to continue for 15 minutes prior to measuring relative fluorescence. B. Immunoblot analysis of protein prepared from the cells (c) and media (m). Equal volumes were loaded in each well. The size markers represent 36 kDa and 40 kDa (note: the ladder used runs approximately 10 kDa higher on this particular type of SDS-PAGE gel, thus the estimated sizes of cytosolic and secreted xylanase are 26 kDa and 28 kDa, respectively). Wild type (WT, cc3395) is also included for comparison. C and D. Extracellular xylanase activity (D) and cell concentration (C) were monitored over the course of four days. Three biological replicates (a–c) of ble2A-SP-xyn1 and the parental strain, cc3395, were inoculated at 0.8×10∧6 cells per ml and allowed to grow to saturation. Xylanase activity is represented as rate of accumulation of the fluorogenic xylanase product DiFMU in µmol/min/ml of culture media. Xylanase reactions contain 45 µls of culture media. Data points represent averages of triplicate measurements.

To determine Xyn1 localization, xylanase activity analysis was performed on the intracellular and secreted protein fractions for the Ble2A-Xyn1 and Ble2A-SP-Xyn1 transgenic lines. As expected, over 80% of the total xylanase activity was found in the intracellular fraction for strain Ble2A-Xyn1 (Figure 5A). However, almost 95% of xylanase activity was found in the culture media for the Ble2A-SP-Xyn1 strain (Figure 5A). Immunoblots performed on the secreted and intracellular fractions demonstrate that the majority of Xyn1 was found in the intracellular fraction (c) for the Ble2A-Xyn1 strain, while the majority of Xyn1 was found in the media (m) in Ble2A-SP-Xyn1 (Figure 5B). Importantly, the expression and secretion of SP-Xyn1 was so robust that we could detect the protein by immunoblot analysis without prior concentration of the media or precipitation of the secreted proteins. Interestingly, the secreted form of Xyn1 had a slower mobility by SDS-PAGE than cytosolic Xyn1, possibly due to post-translational modification (see below).

To further investigate secreted xylanase activity, we performed a time course with 50 ml TAP cultures of three biological replicates (a–c) of the ble2A-SP-Xyn1 transgenic strain and the parental strain. Growth rates as measured by cell concentration and secreted xylanase activity were monitored for four days as cultures grew to saturation (Figure 5C, D). Expression and secretion of Xyn1 did not significantly affect the growth rate of the transgenic line compared to the parental strain (Figure 5C). Xylanase activity in the culture media increased over the time course as the cultures grew to saturation (Figure 5D).

Together, these data show that a recombinant xylanase enzyme can be properly targeted for secretion when fused to an endogenous secretion signal and placed downstream of Ble-2A. Furthermore, the secreted enzyme accumulates in the media and is active.

### Purification and LC-MS/MS analysis of cytoplasmic and secreted Xyn1

Interestingly, secreted Xyn1 migrates slower during SDS-PAGE compared to cytoplasmic Xyn1 (Figure 5B). Several reasons could explain this apparent mobility shift, including failure to cleave the secretion signal peptide or post-translation modification of the secreted or cytoplasmic proteins. To investigate this further, cytoplasmic Xyn1 was purified from Ble2A-Xyn1 cells, while secreted Xyn1 was purified from the media of Ble2A-SP-Xyn1 cultures using anti-FLAG resin (Figure S1A). A xylanase activity assay was performed on purified cytoplasmic Xyn1 and secreted Xyn1. 50 ng of purified protein in PBS was compared to PBS alone (0) or 1 mU of commercial *A. niger* Xylanase. Purified secreted and cytoplasmic Xyn1 (50 ng) displayed similar activities that were close to the activity of 1 mU *A. niger* xylanase (Figure S1C). Interestingly, the protein concentration of *A. niger* Xylanase is 66 µg/ml according to Bradford Assay (BioRad, Hercules, CA). At 295 mU/ml, 3.4 µls (1 mU) was added to the assay. Therefore, 1 mU of activity corresponds to 224 ng of protein. This is compared to the 50 ng of algal-expressed Xyn1. However, we did not determine nor is it clear how pure the *A. niger* Xylanase protein actually is.

As mentioned above, one possible explanation for the slower mobility of secreted Xyn1 is that the Ars1 signal peptide is not properly cleaved upon secretion. To address this, purified cytoplasmic and secreted Xyn1 were subjected to liquid chromatography tandem mass spectrometry (LC-MS/MS) analysis. Each sample was digested with trypsin or chymotrypsin prior to LC-MS/MS analysis and the results are summarized in Figure S1D. For cytoplasmic Xyn1, peptides were identified that covered 97% of the predicted amino acid sequence (234/241 AA). For secreted Xyn1, peptides were identified that covered 89% of the SP-Xyn1 sequence (244/273 AA). Peptides corresponding to the ars1 signal peptide were not identified by LC-MS/MS, indicating that the signal peptide was indeed properly cleaved during transit into the endoplasmic reticulum and secretion from the cell.

The mobility difference between cytosolic and secreted Xyn1 could also be due to differences in post-translational modification. LC-MS/MS analysis revealed that cytosolic Xyn1 is phosphorylated on three separate amino acids, serine 12 or 13, threonine 129 or 131, and on serine 256 of the FLAG tag (Figure S1D, blue amino acids). We did not detect phosphorylated peptides in the secreted Xyn1 sample. However treatment of cytosolic Xyn1 with either protein phosphatase 1 (NEB) or calf intestinal alkaline phosphatase (NEB) failed to cause a noticeable change in mobility (data not shown). Often, secreted proteins are modified by the addition of sugars. To investigate whether secreted Xyn1 is glycosylated, we tested the ability of the purified protein to bind to an array of seven fluorescently-labeled lectins (Vector Lab Lectin Kit I, FLK-2100, Burlingame, CA) in an ELISA assay. However, secreted Xyn1 was unable to bind to any of the seven lectins tested (data not shown). Finally, we incubated secreted Xyn1 with a cocktail of degylcosidases: endo-α-N-acetylgalactosaminidase, glucosaminidase, N-glycosidase F, neuraminidase (Glycoprotein Deglycosylation Kit, Calbiochem, Darmstadt, Germany). Again, we did not see evidence of a change in SDS-PAGE mobility following deglycosidase treatment (data not shown). However, when we immunoprecipitated the intracellular fraction of SP-Xyn1 from Ble2A-SP-Xyn1 cell lysates, we were able to identify a small fraction of protein that ran at a lower molecular weight than the secreted form (Figure S1B, see band marked by ‘*’). This could possibly represent newly synthesized protein prior to, or in the early stages of, post-translational modifications/glycosylation.

## Discussion

*C. reinhardtii* is a valuable eukaryotic model organism for the study of specialized processes such as photosynthesis, flagella function, circadian rhythms, and photoreception. In addition, it has gained recent attention as a bioreactor for the production of recombinant proteins and novel metabolites [12]–[14], [25], [58]. However, a major obstacle has been poor expression of heterologous genes from the nuclear genome. Here we describe a process to achieve robust expression of mature recombinant proteins from the nuclear genome. This was achieved by transcriptionally fusing a recombinant gene to a selection marker gene resulting in robust expression of the recombinant protein. By placing the FMDV 2A sequence between these coding regions a mature recombinant protein was produced. We show that FMDV 2A is properly processed by the microalga and that strains transformed with *ble2A-GFP* are zeocin resistant and accumulate monomeric, cytosolic GFP that is easily detectable by in-gel fluorescence analysis (Figure 2A), fluorescent microscopy (Figure 2B), immunoblotting and flow cytometry (data not shown). Likewise, strains transformed with *ble2A-xyn1* express functional Ble as well as monomeric Xyn1 that is highly active and easily detectable by immunoblotting and xylanase activity assays (Figure 3). We compared our ble2A expression vector to the traditional expression strategy: co-transformation of the unfused monomeric transgene with the selection marker on separate ORFs under the control of distinct promoters. We demonstrate that linking *xyn1* expression to *ble* expression on the same ORF through the 2A self-cleaving sequence led to a dramatic 100 fold increase in xylanase activity in cells lysates compared to the traditional expression vector (Figure 4B). We further demonstrate that this ble2A expression vector can be used to target recombinant proteins for secretion by placing a signal peptide after the 2A sequence and in front of the xylanase coding region, resulting in high levels of xylanase accumulation in the culture media (Figure 5).

The Ble resistance protein functions by antibiotic sequestration rather than enzymatic inactivation, so that only cells expressing relatively high levels of the Ble protein survive zeocin selection. Indeed nuclear transformations of *C. reinhardtii* with the hygromycin B resistance enzyme *aph7″*, typically yield 10 to 100-fold more colonies than transformations with *ble*, presumably because of the requirement for high levels of ble protein production for resistance. Thus, we believe the reason we see such high levels of transgene expression using the Ble2A expression vector is due to the nature of the initial *ble* selection, where only robust expression of ble confers survival on zeocin plates. Because recombinant xylanase expression is directly linked to ble expression on the same open reading frame, the selection of high ble expression likely ensures the selection of high xylanase expression. Furthermore, continued selection on zeocin ensures that the transgene, both ble and the linked xylanase protein, will not be silenced.

As described above, our ble2A expression vector offers several advantages over traditional nuclear expression vectors: 1) it enables the selection of transgenic lines that express a gene-of-interest at significantly higher levels, 2) it leads to expression of near native proteins, modified by a single proline addition on the N-terminus, and 3) it can be used to target proteins for secretion, and likely other sub-cellular locations. Indeed, in other eukaryotes, proteins processed by 2A from polyproteins have been successfully targeted to the cytosol, nucleus, mitochondria, endoplasmic reticulum, Golgi apparatus, plasma membrane and the extra-cellular compartment [47]. Thus, we believe our ble2A expression strategy will become an important tool for both basic research and algal biotechnology. In terms of research applications, the ble2A expression vector linked to a reporter gene can be used for promoter studies and high throughput screening of mutant libraries, for example. It can also be used to over-express endogenous genes for mutant complementation or overexpression studies. Additionally, endogenous genes can be tagged with fluorescent proteins to investigate in vivo localization or in vivo protein-protein interactions.

In terms of biotechnological applications, we show that the ble2A vector can be used to express and accumulate, or secrete, the industrial enzyme xylanase. Together with cellulases and pectinases, xylanases account for 20% of the world's enzyme market [53]. While various microorganisms such as bacteria, yeasts, and filamentous fungi naturally secrete xylanases, heterologous expression of this enzyme would be preferred in cases where only this activity is wanted. Microorganisms that naturally secrete xylanases often also secrete other cellulose degrading enzymes, such as cellulases, whose activities could have adverse effects, on paper manufacturing for example [54]. This ble2A expression system may also prove valuable for algal metabolic engineering, an area of research gaining attention recently due to microalgae's potential as a renewable source of biofuels and biochemicals. Heterologous enzymes could be targeted to the chloroplast or endoplasmic reticulum, sites of hydrocarbon synthesis. The FMDV 2A sequence could also be used to co-express a transgene with an endogenous gene of interest. Indeed, we constructed an Arg7-2A-xyn expression vector that rescued an arginine mutant and also led to detectable monomeric xylanase accumulation (data not shown).

For microalgae to be economically successful as a biotechnology platform, high, stable, and consistent levels of recombinant protein accumulation will be required. We have developed a novel microalgal nuclear expression vector that enables the selection of high recombinant protein expressing lines, and we believe this tool will prove valuable for the future of algal transgenics and biotechnology.

## Materials and Methods

### Construction of plasmids

All plasmids were built on the pBluescript II backbone. The two nuclear promoters used in this study were P_AR1_, hsp70A/rbcs2 promoter containing the first intron of rbcs2 (Figure S2), and P_AR4_, the hsp70A promoter fused to four copies of the first rbcs2 intron and then to the rbcs2 promoter (Figure S3). Both promoters were cloned into pBS II as XbaI/NdeI fragments. The *sh-ble-GFP* direct fusion gene [35] was cloned behind P_AR1_ as an NdeI/BamHI fragment (Figure S5). The FMDV 2A sequence was fused to the end of *sh-ble* by PCR using a long reverse primer encoding for the codon-optimized 2A sequence. *ble-2A* was then cloned behind P_AR4_ as an NdeI/XhoI fragment (Figure S6). The *Trichoderma reesei xylanase 1* gene was codon optimized for *C. reinhardtii* nuclear expression and synthesized by DNA 2.0 (Figure S7). GFP or Xyn1 was cloned behind P_AR4_::Ble2A as XhoI/BamHI fragments. The ars1 secretion signal peptide was generated as a SalI/XhoI fragment and ligated into pBle2A-Xyn1 that was linearized by XhoI (Figure S7). Xyn1 was also cloned directly behind P_AR4_ as an NdeI/BamHI fragment to generate the pHyg Xyn1 vector. The hygromycin B resistance gene was under the control of the beta-tubulin promoter [57]. All constructs contained the rbcs2 3′ UTR (Figure S4).

### *C. reinhardtii* strains, transformations and growth conditions

The cell-walled *C. reinhardtii* strain used in this study was cc1690, the cell wall-deficient strain was cc3395. Both were transformed by electroporation. For cc1690 electroporations, cells were grown to 3–6×10^6^ cells/ml in TAP (Tris–acetate–phosphate) medium [59] at 23 degrees C under constant illumination of 5000 lux on a rotary shaker. Cells were harvested by centrifugation and resuspended in TAP medium supplemented with 40 mM sucrose at a concentration of 3×10^8^ cells/ml. 250 µls of cells were incubated with 300–1000 ng of digested transformation plasmid for 5–10 minutes on ice in a 4 mm cuvette. An exponential electric pulse of 2000 V/cm was applied to the sample using a GenePulser XCell™ (BioRad, Hercules, CA) electroporation apparatus. The capacitance was set at 25 µF and no shunt resistor was used. Cells were recovered for 18 hours in 10 mls of TAP/40 mM sucrose and then plated to two TAP/agar plates supplemented with the appropriate antibiotic. Electroporations of the cc3395 were identical, except that the resistance was set to 200 and arginine was also added to the media at 100 µg/ml. Note: the transformation plasmids were either linearized with a single restriction enzyme that cut only once, or double digested on either side of the expression cassette. We saw increased transformation efficiency, and increased co-transformation efficiency with pHyg Xyn1, when the expression cassettes were double digested, similar to [60].

### PCR screens for the identification of gene positive transformants

The stable integration of GFP or xylanase was determined by PCR analysis of cell lysates, as described previously (58). For *GFP* gene positive screens, the oligonucleotides 5- CAAGTCCGCCATGCCCGAGG-3 and 5′- CGGCAGCGGTGACGAACTCC-3′ were used. For *xyn1* gene positive PCR screens, the oligonucleotides 5′- AACTCGAGGCGAGCATTAACTACGACC-3′ and TTGGATCCTTAGGACTTGTCGTCGTCGTC-3′ were used.

### GFP analysis – in-gel fluorescence and microscopy

#### In-gel fluorescence analysis

Representative ble-GFP and ble2A-GFP clones were grown in liquid to saturation. Cells were harvested by centrifugation and resuspended in SDS-PAGE loading buffer containing no β-mercaptoethanol, causing cell lysis. Cell lysates representing equal volumes of cells were loaded onto an SDS-PAGE gel that contained no β-mercaptoethanol. Fluorescent signals were visualized using a Berthold Night Owl CCD camera, model LB 981 (Berthold Technologies, Bad Wildbad, Germany), equipped with a blue light source and an emission filter of 535 nm RDF 45 (Omega Optical, Brattle, VT).

#### Fluorescence microscopy

Representative ble-GFP and ble2A-GFP clones were grown in liquid to mid-log phase. Images were captured with an Applied Precision Spectris optical sectioning microscope system equipped with an Olympus IX70 microscope, an Olympus Plan Apo 100× oil immersion objective (NA 1.4), a Photometrics Cool SNAP HQ digital camera and DeltaVision standard fluorescence filters: FITC for GFP visualization (excitation: blue 490/20 nm; emission: green 528/38 nm). Using SoftWoRx software, the brightness and contrast were adjusted, setting the area outside of cells to be background; images were saved as TIFF files.

### Xylanase activity assays and immunoblotting

#### Xylanase assay on total soluble protein

Cells were grown in TAP until late log phase, harvested by centrifugation at 5000×*g*, and resuspended in PBS, 0.1% Tween, 1 mM PMSF. Cells were lysed by sonication and spun at 12,000×*g* for 10 minutes. Total soluble protein was retained and protein concentration was measured using BioRad Protein Reagent as per the manufacturer's instructions. Xylanase activity was measured using the EnzChek® Ultra Xylanase Activity Kit (Life Technologies, Carlsbad, CA). Hydrolysis of the fluorogenic substrate 6,8-difluoro-4-methylumbelliferyl β-d-xylobioside (DiFMUX_2_) by xylanase leads to increased fluorescence at excitation/emission 385/455 nm over time [55]. Cell lysates were diluted to 1 µg/µl in xylanase reaction buffer (100 mM sodium acetate, pH 4.6) and 50 µls was transferred in duplicate or triplicate to a black 96-well plate (Costar, Lowell, MA). The activity of algal-expressed Xyn1 was compared to commercial *Aspergillus niger* Xylanase (Megazyme, Wicklow, Ireland). 2.5 µg of xylanase substrate was added to xylanase-containing samples. Reactions were incubated at 42 degrees C and fluorescence readings were taken every 5 minutes for 30–60 minutes using a Tecan plate reader. (Tecan Infinite® M200 PRO, Männedorf, Switzerland). Product formation rates (µmol/min) were calculated as per the manufacturer's instructions.

#### Xylanase activity assay on the culture media

To assay for secreted xylanase activity in the culture media, the cultures were gently spun at 2500×*g* and media was retained. 5 µls of 10× xylanase buffer was diluted in 45 µls of cell-free media in triplicate in a black 96 well plate. 2.5 µg of xylanase substrate (EnzChek® Ultra Xylanase Activity Kit) was added to xylanase-containing samples, reactions were incubated at 42 degrees C and fluorescence readings were taken every 5 minutes for 30–60 minutes. Product formation rates (µmol/min/ml) were calculated as per the manufacturer's instructions.

#### Determining localization of xylanase activity – cells vs media

*Ble2A-xyn1* and *Ble2A-SP-xyn1* cultures were grown in triplicate in TAP media supplemented with arginine for four days. 500 µls of each culture was transferred to microfuge tubes and cells were gently pelleted at 2,500×*g* for 5 minutes. The cell-free media were transferred to clean tubes and represent the secreted protein fraction (m). The pelleted cells were resuspended in 500 µls of fresh TAP media and represent the intracellular protein fraction (c). 50 µls of each fraction was incubated in triplicate with 25 µls of BugBuster® Protein Extraction Reagent (Novagen, Darmstadt, Germany) for 5 minutes at room temperature in a black 96 well plate. This step enabled lysis of the cells in the (c) fraction (data not shown) and release of intracellular Xyn1. 10 µls of the 10× xylanase buffer and 2.5 µg of the xylanase substrate were added to each protein fraction and xylanase activity was monitored by increase in fluorescence over time in a Tecan plate reader. The average fluorescence, which represents xylanase activity for the secreted and intracellular protein fractions, was added together to determine total xylanase activity for the Ble2A-xyn1 and Ble2A-sp-xyn1 strains. The average fluorescence for the intracellular or secreted fractions were divided by total fluorescence and the calculated xylanase activity is shown as a percentage of total activity. Immunoblots were performed on the secreted and intracellular fractions; the samples were diluted with 4×Laemmli loading buffer and 30 µls were analyzed by SDS-PAGE.

#### Immunoblotting

TSP was denatured by the addition of SDS–PAGE loading buffer (Laemmli) followed by incubation at 95 degrees C for 3 min. Proteins were separated on 12% SDS–PAGE gels at 120–150 volts and transferred to nitrocellulose membrane at 200 mAmps for 1.5 h. After blocking with 5% milk, membranes were probed with an anti-FLAG monoclonal antibody conjugated to alkaline phosphatase (A9469; Sigma, St. Louis, MO).

### Protein purification and mass spectrometry

For the purification of cytoplasmic Xyn1, total soluble protein from 500 ml cultures was incubated with 0.5 mL of anti-FLAG M2 resin (Sigma) in binding buffer (50 mM Tris pH 8.0, 400 mM NaCl, 0.1% Tween 20) and rotated end-over-end at 4 degrees C for 4 h. For secreted Xyn1 purification, 500 mls of cell free media was incubated with 1 ml of anti-FLAG resin and rotated end-over-end at 4 degrees C for 4 hours. The anti-FLAG beads were collected by centrifugation and added to a Bio-Rad Econo-pac column and washed extensively with binding buffer. The protein was eluted from the resin using 100 mM glycine pH 3.5, 400 mM NaCl and neutralized with Tris pH 7.9 to final a concentration of 50 mM. Purified protein was concentrated and the buffer was exchanged to PBS using an Amicon Ultra centrifugal filter with a molecular weight cut-off of 3 kDa (Millipore, Billerica, MA, USA). Protein concentrations were determined using the BioRad Protein Reagent (Bio-Rad) with bovine serum albumin as a standard, and samples were analyzed by SDS-PAGE.

#### Mass spectrometry

Purified cytoplasmic and secreted Xyn1 protein solutions (35 ng/µl in 50 mM Hepes buffer, pH 7.2) were reduced and alkylated using 2 mM Tris (2-carboxyethyl) phosphine (Fisher, AC36383) at 65°C for 5 minutes and 5 mM iodoacetamide (Fisher, AC12227, Pittsburgh, PA) at 37°C in dark for 30 minutes, respectively. Each sample was split into 2 equal halves and digested with 0.2 µg trypsin (Roche, Basel, Switzerland) and chymotrypsin (Roche) at 37°C overnight.

Automated 2D nanoflow LC-MS/MS analysis was performed using LTQ tandem mass spectrometer (Thermo Electron Corporation, San Jose, CA) employing automated data-dependent acquisition. An Agilent 1100 HPLC system (Agilent Technologies, Wilmington, DE) was used to deliver a flow rate of 500 nl min–1 to the mass spectrometer through a splitter. Chromatographic separation was accomplished using a 3 phase capillary column. Using an in-house constructed pressure cell, 5 µm Zorbax SB-C18 (Agilent) packing material was packed into a fused silica capillary tubing (200 µm inner diameter (ID), 360 µm OD, 10 cm long) to form the first dimension RP column (RP1). A similar column (200 µm ID, 5 cm long) packed with 5 µm polysulfoethyl (PolyLC) packing material was used as the SCX column. A zero dead volume 1 µm filter (Upchurch, M548, Oak Harbor, WA) was attached to the exit of each column for column packing and connecting. A fused silica capillary (200 µm ID, 360 µm OD, 20 cm long) packed with 3.5 µm Zorbax SB-C18 (Agilent) packing material was used as the analytical column (RP2). One end of the fused silica tubing was pulled to a sharp tip with the ID smaller than 1 µm using a laser puller (Sutter P-2000) as the electro-spray tip. The peptide mixtures were loaded onto the RP1 column using the same in-house pressure cell. To avoid sample carry-over and keep good reproducibility, a new set of three columns with the same length was used for each sample. Peptides were first eluted from RP1 column to SCX column using a 0 to 80% acetonitrile gradient for 150 minutes. Then peptides were fractionated by the SCX column using a series of 8 step salts (10 mM, 15 mM, 20 mM, 30 mM, 50 mM, 70 mM, 100 mM, and 1 M ammonium acetate for 20 minutes), followed by high resolution reverse phase separation using an acetonitrile gradient of 0 to 80% for 120 minutes.

Raw data were extracted and searched using Spectrum Mill (Agilent, version A.03.02.060b). MS/MS spectra with a sequence tag length of 1 or less were considered poor and were discarded. The filtered MS/MS spectra were searched against a database containing the cytoplasmic and secreted Xyn1 protein sequences and common contaminants including trypsin and keratin. The enzyme parameter was limited to full tryptic or chymotryptic peptides with a maximum mis-cleavage of 2. All other search parameters were set to SpectrumMill's default settings (carbamidomethylation of cysteines, ±2.5 Da for precursor ions, ±0.7 Da for fragment ions, and a minimum matched peak intensity of 50%). S/T/Y phosphorylation, oxidized-Methionine and pyroGlutamate were defined as variable modifications. A maximum of 2 modifications per peptide was used. Search results for individual spectra were validated using the filtering criteria listed in Table 1.

**Table 1:** Filtering Criteria for autovalidation of database search results.

mode	Protein score	1+ peptide	2+ peptide	3+ peptide
Protein Details	>20	>11, >50%	>11, >50%	>13, >50%
Peptide	NA	>13, >50%	>13, >50%	>15, >50%

## Supporting Information

Figure S1 **Purification of secreted and intracellular Xyn1.** A. Intracellular Xyn1 (cyt) was purified from lysates of cells transformed with *ble2A-xyn1*, while secreted Xyn1 (sec) was purified from the cell-free media isolated from a culture containing cells transformed with *ble2A-SP-xyn1*. Protein samples from the purification - input, flow through, and purified protein (elution) – were subjected to immunoblot analysis. Note: protein samples were run out on a Tricine SDS-PAGE gel. The presence of Tricine slows the mobility of the protein marker by approximately 8–10 kDa. B. Intracellular SP-Xyn1 from the *ble2A-SP-xyn1* strain was immunoprecipitated from cell lysates (intracellular, left) with anti-FLAG resin. A higher mobility band (‘*’) was detected in the intracellular fraction that was not seen in the secreted fraction that was immunoprecipitated from the culture media (secreted, right) C. Xylanase activity was measured for 50 ng of purified proteins. 1 mU of commercial xylanase was used as a control and for comparison. Relative fluorescence was measured 10 minutes after incubation with the substrate. D. Results from LC-MS/MS analysis of purified cytosolic Xyn1 and secreted Xyn1 digested with either trypsin or chymotrypsin. Amino acids in black were not identified by mass spectrometry. Amino acids in blue were identified as phosphorylated (either Ser12 or Ser13, either Thr129 or Thr131, and Ser224). Underlined amino acids indicate the ars1 secretion signal peptide.
(TIF)

Figure S2 **P_AR1_ sequence.** The sequence of P_AR1_, which contains the hsp70A promoter enhancer element, the rbcs2 promoter and 5′ UTR, and one copy of the rbcs2 intron 1.
(TIF)

Figure S3 **P_AR4_ sequence.** The sequence of P_AR4_, which contains the hsp70A promoter enhancer element, four parallel copies of the rbcs2 intron 1, and the rbcs2 promoter and 5′ UTR.
(TIF)

Figure S4 **Rbcs2 3′ UTR sequence.** The sequence of the rbcs2 3′ UTR terminator, which was used in all of the nuclear expression vectors in this study.
(TIF)

Figure S5 **Ble-GFP sequence.** The sequence of ble-GFP, a direct fusion of *ble* and *GFP* containing one copy of the rbcs2 intron 1 inserted into the ble coding sequence.
(TIF)

Figure S6 **Ble-2A sequence.** The ble sequence is identical to that in ble-GFP, containing one copy of the rbcs2 intron 1. The FMDV 2A coding sequence was codon-optimized and fused to the end of the ble gene by PCR.
(TIF)

Figure S7 **SP-Xyn1 sequence.** *T. reesei xylanase 1* was codon-optimized for *C. reinhardtii* nuclear expression and synthesized as an XhoI/BamHI fragment. The *C. reinhardtii ars1* secretion sequence was inserted between *ble2A* and *xyn1* as a SalI/XhoI fragment.
(TIF)
