# Supplementary figures and images for: Robust Expression and Secretion of Xylanase1 in Chlamydomonas reinhardtii by Fusion to a Selection Gene and Processing with the FMDV 2A Peptide
Source: PLoS One. 2012 Aug 24;7(8):e43349. doi: 10.1371/journal.pone.0043349 (PMC3427385; doi:10.1371/journal.pone.0043349)

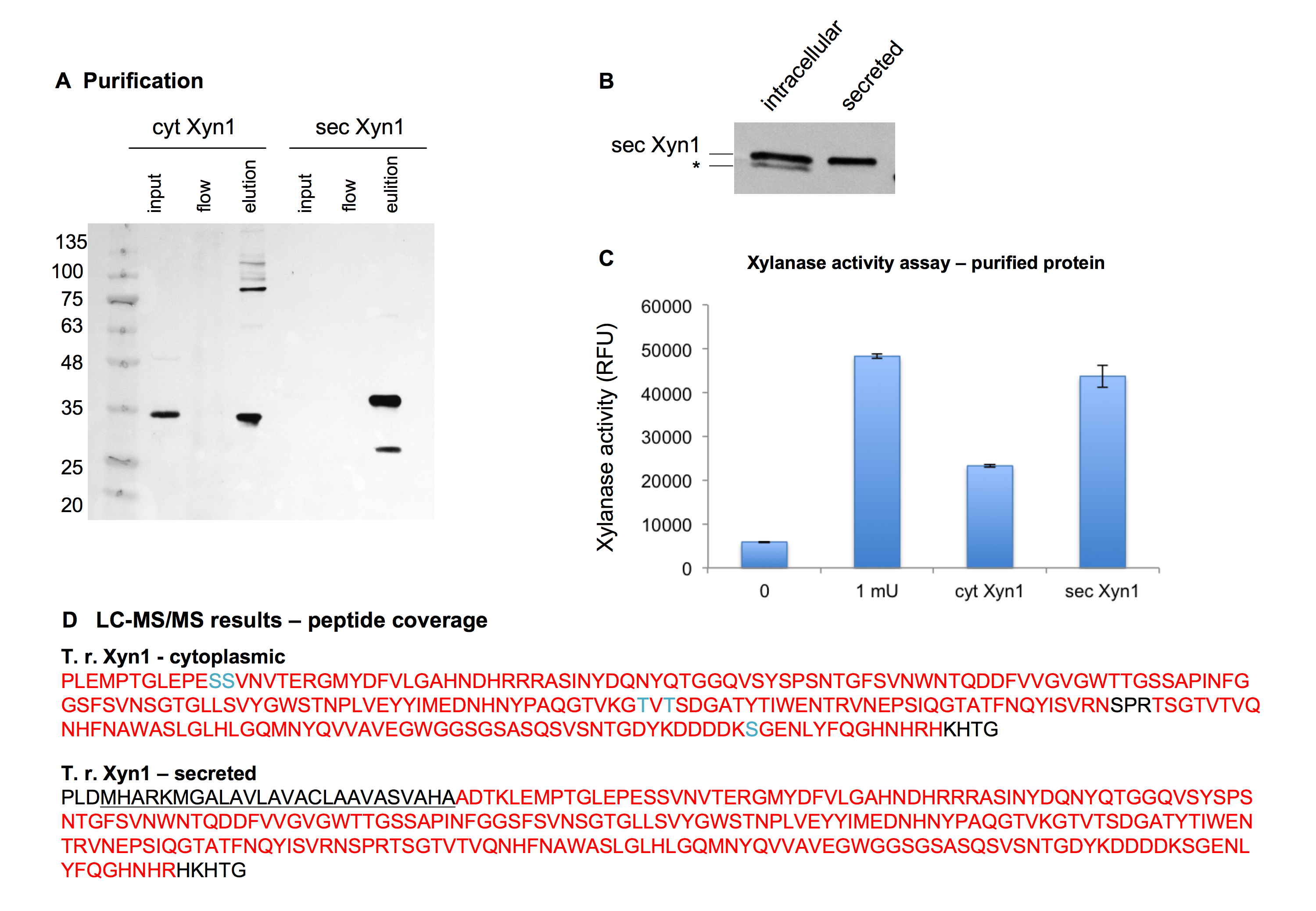

Supplement: Figure S1 — Purification of secreted and intracellular Xyn1. A. Intracellular Xyn1 (cyt) was purified from lysates of cells transformed with ble2A-xyn1, while secreted Xyn1 (sec) was purified from the cell-free media isolated from a culture containing cells transformed with ble2A-SP-xyn1. Protein samples from the purification - input, flow through, and purified protein (elution) – were subjected to immunoblot analysis. Note: protein samples were run out on a Tricine SDS-PAGE gel. The presence of Tricine slows the mobility of the protein marker by approximately 8–10 kDa. B. Intracellular SP-Xyn1 from the ble2A-SP-xyn1 strain was immunoprecipitated from cell lysates (intracellular, left) with anti-FLAG resin. A higher mobility band (‘*’) was detected in the intracellular fraction that was not seen in the secreted fraction that was immunoprecipitated from the culture media (secreted, right) C. Xylanase activity was measured for 50 ng of purified proteins. 1 mU of commercial xylanase was used as a control and for comparison. Relative fluorescence was measured 10 minutes after incubation with the substrate. D. Results from LC-MS/MS analysis of purified cytosolic Xyn1 and secreted Xyn1 digested with either trypsin or chymotrypsin. Amino acids in black were not identified by mass spectrometry. Amino acids in blue were identified as phosphorylated (either Ser12 or Ser13, either Thr129 or Thr131, and Ser224). Underlined amino acids indicate the ars1 secretion signal peptide. (TIF) [file pone.0043349.s001.tif]

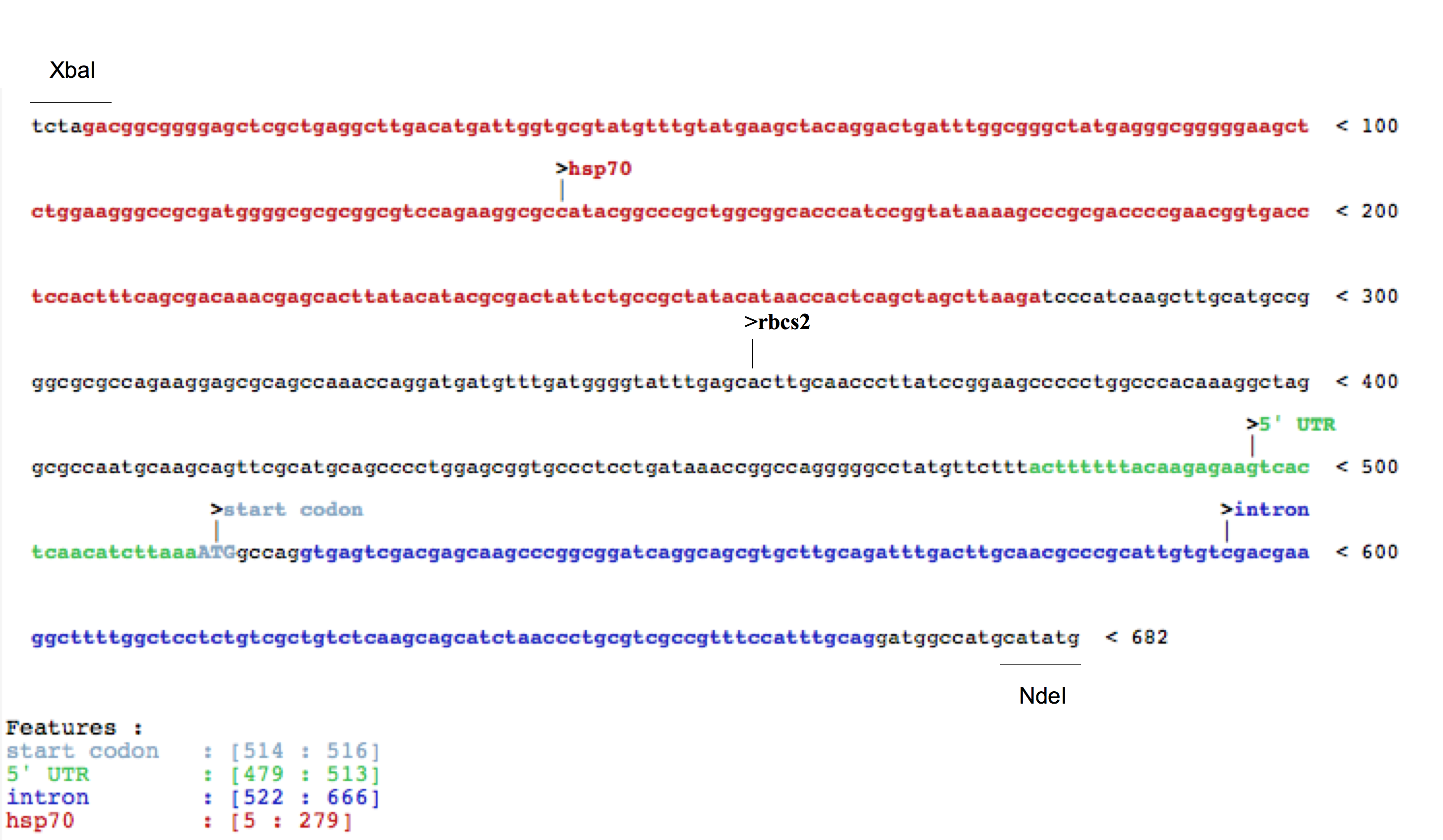

Supplement: Figure S2 — PAR1 sequence. The sequence of PAR1, which contains the hsp70A promoter enhancer element, the rbcs2 promoter and 5′ UTR, and one copy of the rbcs2 intron 1. (TIF) [file pone.0043349.s002.tif]

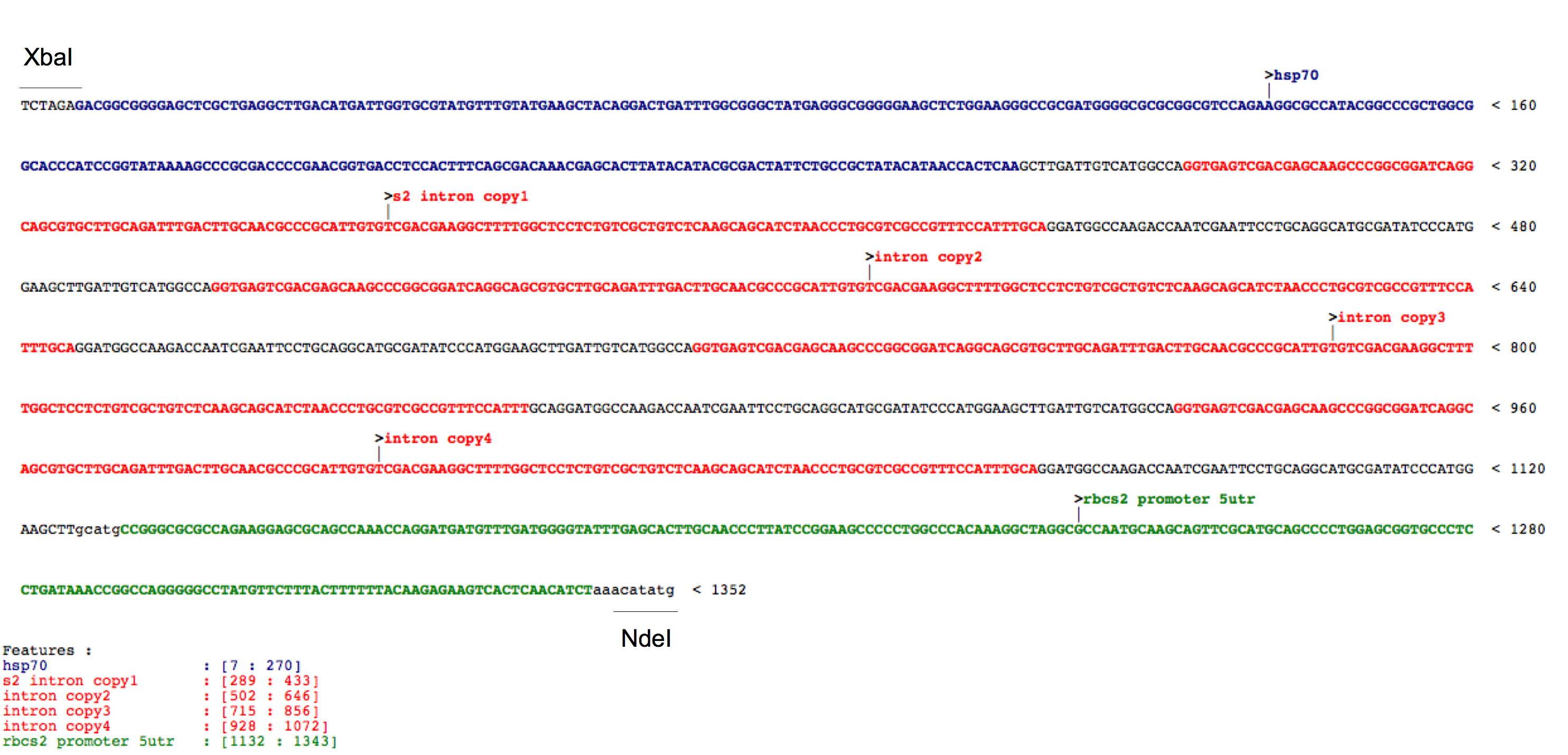

Supplement: Figure S3 — PAR4 sequence. The sequence of PAR4, which contains the hsp70A promoter enhancer element, four parallel copies of the rbcs2 intron 1, and the rbcs2 promoter and 5′ UTR. (TIF) [file pone.0043349.s003.tif]

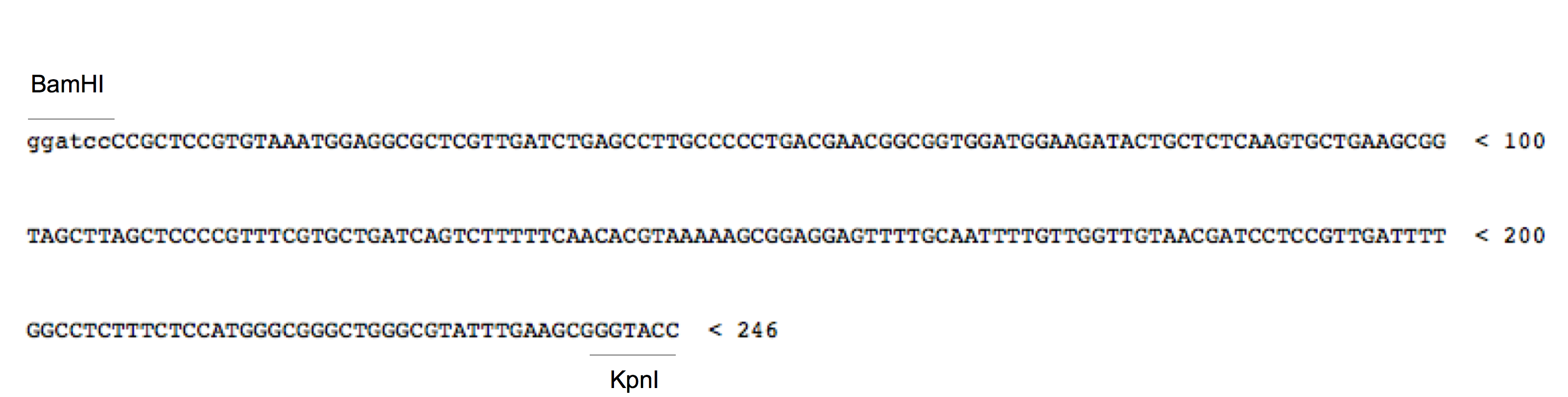

Supplement: Figure S4 — Rbcs2 3′ UTR sequence. The sequence of the rbcs2 3′ UTR terminator, which was used in all of the nuclear expression vectors in this study. (TIF) [file pone.0043349.s004.tif]

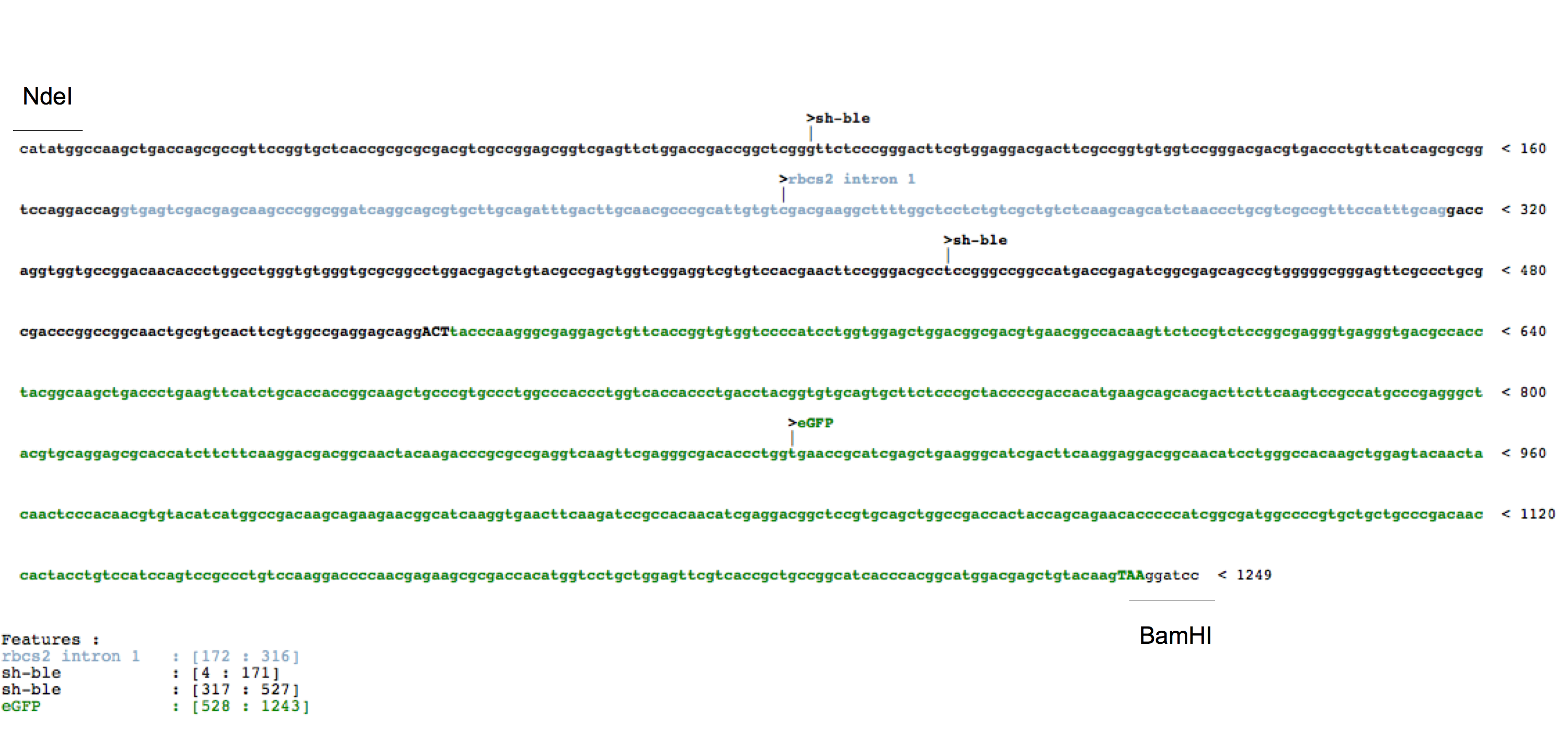

Supplement: Figure S5 — Ble-GFP sequence. The sequence of ble-GFP, a direct fusion of ble and GFP containing one copy of the rbcs2 intron 1 inserted into the ble coding sequence. (TIF) [file pone.0043349.s005.tif]

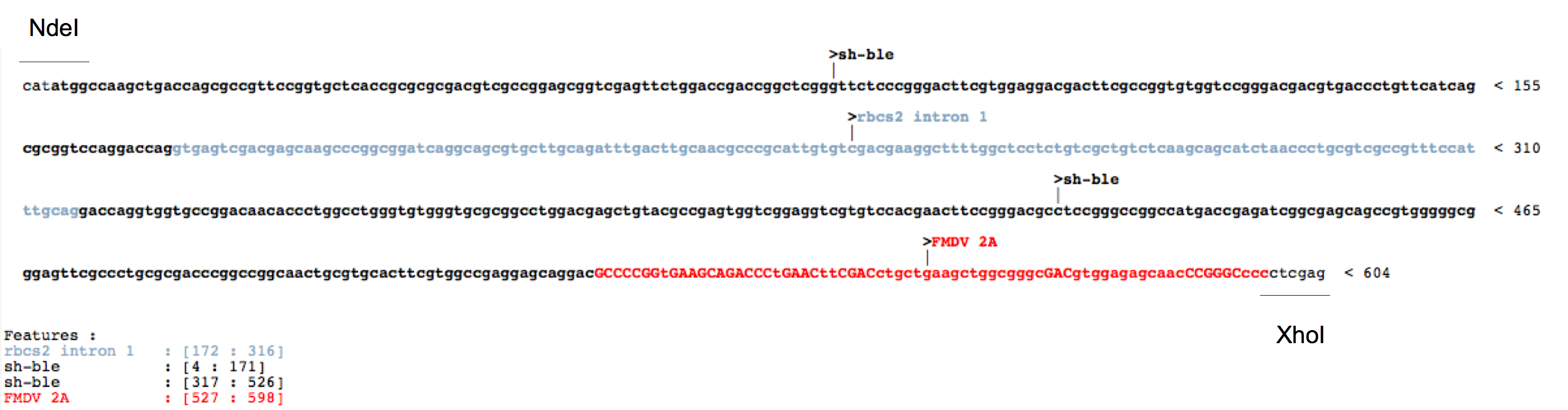

Supplement: Figure S6 — Ble-2A sequence. The ble sequence is identical to that in ble-GFP, containing one copy of the rbcs2 intron 1. The FMDV 2A coding sequence was codon-optimized and fused to the end of the ble gene by PCR. (TIF) [file pone.0043349.s006.tif]

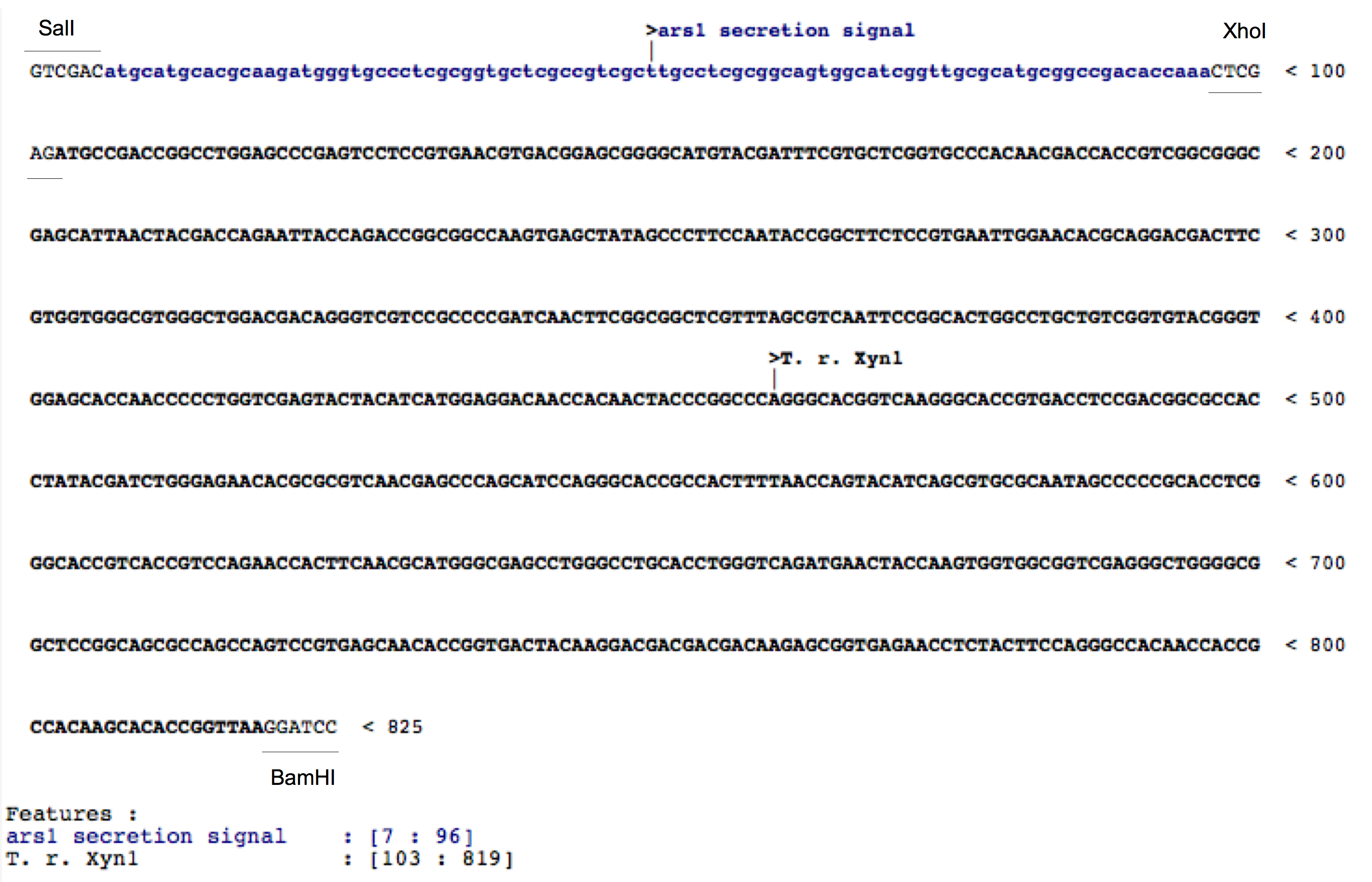

Supplement: Figure S7 — SP-Xyn1 sequence. T. reesei xylanase 1 was codon-optimized for C. reinhardtii nuclear expression and synthesized as an XhoI/BamHI fragment. The C. reinhardtii ars1 secretion sequence was inserted between ble2A and xyn1 as a SalI/XhoI fragment. (TIF) [file pone.0043349.s007.tif]
